# Mental Rotation of Faces in Healthy Aging and Alzheimer's Disease

**DOI:** 10.1371/journal.pone.0006120

**Published:** 2009-07-02

**Authors:** Cassandra A. Adduri, Jonathan J. Marotta

**Affiliations:** Perception and Action Lab, Department of Psychology, University of Manitoba, Winnipeg, Manitoba, Canada; Centre de Recherches su la Cognition Animale - Centre National de la Recherche Scientifique and Université Paul Sabatier, France

## Abstract

**Background:**

Previous research has shown that individuals with Alzheimer's disease (AD) develop visuospatial difficulties that affect their ability to mentally rotate objects. Surprisingly, the existing literature has generally ignored the impact of this mental rotation deficit on the ability of AD patients to recognize faces from different angles. Instead, the devastating loss of the ability to recognize friends and family members in AD has primarily been attributed to memory loss and agnosia in later stages of the disorder. The impact of AD on areas of the brain important for mental rotation should not be overlooked by face processing investigations – even in early stages of the disorder.

**Methodology/Principal Findings:**

This study investigated the sensitivity of face processing in AD, young controls and older non-neurological controls to two changes of the stimuli – a rotation in depth and an inversion. The control groups showed a systematic effect of depth rotation, with errors increasing with the angle of rotation, and with inversion. The majority of the AD group was not impaired when faces were presented upright and no transformation in depth was required, and were most accurate when all faces were presented in frontal views, but accuracy was severely impaired with any rotation or inversion.

**Conclusions/Significance:**

These results suggest that with the onset of AD, mental rotation difficulties arise that affect the ability to recognize faces presented at different angles. The finding that a frontal view is “preferred” by these patients provides a valuable communication strategy for health care workers.

## Introduction

Imagine trying to find a familiar face as you walk into a crowded party. As you scan the room, you recognize your friend fairly easily even though she may not be looking directly at you. When you finally make your way towards her and engage in a conversation, she may turn away for a second. Even though the act of your friend turning results in different retinal input, you are not led to believe that you are now speaking to a different person. Similarly, at this same party, you may put your glass down on a table, and despite looking at it from a different angle when you pick it up, you still recognize it as your glass. This success in recognizing people and objects from different viewpoints relies on robust image representations that are resilient to large changes in retinal inputs. How one derives these invariant representations is one of the crucial questions in vision science. Despite the large amount of research in this field, the question of whether or not the strength of these representations changes during our lifetime, or is affected by neurodegenerative disorders like Alzheimer's disease (AD), has been largely unstudied.

Previous research has shown that even though we are able to recognize objects and faces from different vantage points, it is not done without cost. Viewpoint-dependent theories of recognition suggest that the viewer must mentally rotate the image to a canonical orientation and the further the presentation angle is from this canonical view, the more recognition times and errors increase [1]–[5]. For face recognition, there is strong evidence that a three-quarter view is the canonical view, as it produces the fastest and most accurate responses [6]–[9]. However it should be noted that the three-quarter view advantage is still being debated, as some have found that a frontal view of a face can show the greatest advantage [10].

In addition to rotations in depth, planar transformations have also been shown to have significant effects on face recognition. Yin (1969) [11] was the first to find that faces were more difficult to recognize when they were inverted, the inversion effect, leading him to conclude that faces are not represented in a face-centered or view-invariant way. Yin also found that face recognition was disproportionately impaired by stimulus inversion when compared to recognition of other objects. This result has been replicated many times and is a standard in the literature [for review, see 12].

The cost of mentally rotating objects appears to be fundamentally affected by aging. Although older viewers consistently show longer reaction times than younger viewers when mentally rotating objects and shapes [13]–[16], it has also been argued that different strategies may be utilized. While young adults utilize a holistic approach when mentally rotating simple shapes, and a piecemeal/parts-based approach when mentally rotating complex shapes, older adults use a holistic approach for both tasks. This strategy may serve to reduce cognitive load with the older adults [16].

In contrast to object rotation, there has been little investigation of the effects of normal and pathological aging on our ability to mentally rotate faces. Only recently has the ability to rotate faces in healthy older individuals been investigated [17]. In a behavioral study using synthetic face stimuli to measure thresholds for detecting differences between similar faces, older and younger adults were not found to differ for faces in identical views but there was a marked impairment in healthy older individuals making matches across a 20° rotation from full-face [17]. This exciting finding highlights the need to extend this research using real images of faces over longer duration periods. Further, the effect that pathological disorders like AD have on the ability to mentally rotate faces has been less studied.

AD is a neurodegenerative disease that progressively destroys a person's memory, and his or her ability to learn and reason, make judgments, communicate, and carry out daily activities. While memory is often what is affected first, it is typically followed by a progressive decline of executive functions, language, perception and visuospatial skills [18], [19]. As the disease progresses towards a more moderate stage, patients may require assistance in everyday tasks. At this stage, patients may be unable to recall names of family members, or their addresses – this memory impairment will continue to worsen as the late stage of the disease begins [20].

A widespread cortical network has been implicated in mental rotation. Cortical activation areas have been found spanning the parietal, prefrontal, occipital, and temporal areas [21]–[24]. Given that AD is associated with extensive damage to many of these same areas [25], [26], it may not be surprising that many people with AD have difficulty mentally rotating shapes and objects [27]–[29]. This is true despite the fact that, at least in the early to moderate stages of the disease, they perform as well as healthy elderly controls on matching tasks that do not require mental rotation [28], [29].

An investigation by Murphy, Kohler, Black and Evans (2000) revealed that AD patients had great difficulty matching identical, non-symmetrical shapes (called Blake shapes) when they were presented at different orientations to one another. In fact, research has shown that as the angular disparity increases between objects, the AD patients' performance is more impaired than that of age-matched controls [27], [29]. Furthermore, participants with mild to moderate AD perform poorly on tasks in which they have to visually identify objects rotated at various rotation angles, provide the canonical orientation, and mentally rotate these same objects [30]. This deficit, called orientation agnosia, can also be seen when AD patients copy simple pictures - their drawings can be rotated, sometimes up to 90° or 180°, when compared to the original [31].

What about the ability of individuals with AD to mentally rotate faces? Is it disproportionately impaired in these individuals? Traditionally, AD studies of face processing have generally concentrated on famous face tasks [32] and those that test new retrieval methods to associate specific faces with names [33]. Although these kinds of studies have revealed that people with AD are impaired at face recognition tasks, they assume that the impairment is primarily the result of a memory deficit. More recently, a study by Lee, Buckley, Gaffan, et al. (2006) revealed that AD participants in early stage of the disease were not impaired when required to choose the odd-one-out of four faces, which could be presented at the same or different angle to the other 3 faces, but were impaired when scenes were presented this way. Although mental rotation difficulties with scenes were observed in this study, the AD participants were not impaired when selecting the “odd” rotated face [34]. It is worth noting, however, that this sample of AD participants was fairly young (mean age 70.14, S.D. = 5.55), and had high MMSE scores (mean = 23.57, S.D. = 3.91), which suggests that these AD patients may have been in very early stages of the disease. Additionally, AD patients had an unlimited amount of time to complete the task and reaction times were not reported in the manuscript. It is quite possible then that a speed-accuracy trade-off may have occurred, and with a time-limited face mental rotation task, difficulties with faces may have been observed.

As a first step in our investigation, we tested the effects of aging on the ability of young adults and older non-neurological controls to complete a time-limited face-matching task in which stimuli could be rotated in depth and/or inverted. We wanted to determine if aging on its own hampers the ability of individuals to mentally rotate faces; if orientation affects young and old participants in the same way and if there was a “preferred” view of the face in which performance was better for each of the groups. Following this, the performance of a subgroup of our “oldest” aged controls was compared to a group of AD patients in order to determine if individuals with AD are disproportionately impaired in their ability to mentally rotate a face.

## Materials and Methods

### 

#### Ethics Statement

Procedures involving experiments on human subjects were done in accord with the ethical standards of the University of Manitoba's Research Ethics Board.

### Experiment #1 – The effects of aging on face processing

#### Participants

All participants gave informed, written consent before beginning the study. Fifteen young adults (7 males, 8 females, mean age = 23.0 years old, S.D. = 4) were recruited from an Introduction to Psychology course at the University of Manitoba and received credit toward a course requirement for their participation. These individuals were right-handed with normal or corrected-to-normal vision. Twelve healthy elderly adults (5 males, 7 females, mean age = 74.9 years old, S.D. = 9) were recruited from the Centre on Aging at the University of Manitoba. All participants were right-handed, had no known neurological problems, did not self-report a depression diagnosis, and had their near visual acuity tested under binocular viewing conditions (see Table 1). No participants self-reported having glaucoma or macular degeneration.

**Table 1:** Information on age, education, MMSE, DRS, and visual acuity scores for the older control group.

Subject	Age	Education (years)	MMSE (raw score)	MMSE (T-score)	DRS (raw score)	DRS (T-score)	Visual acuity	Cataracts
1	63	18	30	57.7	142	59.5	20/30	No
2	75	21	29	56.3	140	56.0	20/30	Yes (right)
3	86	12	29	65.0	138	56.0	20/20	No
4	73	12	30	68.8	144	66.0	20/20	No
5	78	10	29	63.3	140	56.0	20/30	No
6	68	10	30	64.3	139	50.0	20/20	No
7	64	21	30	57.7	141	56.0	20/20	No
8	84	12	29	67.4	138	56.0	20/25	Yes (both)
9	81	11	30	71.7	137	52.0	20/20	No
10	62	12	29	55.9	143	62.5	20/25	No
11	78	9	30	70.0	140	56.0	20/25	Yes (both)
12	87	9	29	65.0	142	66.0	20/30	No

Prior to testing on the computerized face-matching task, all participants over 60 years of age completed two cognitive screening tools: the Mini Mental State Examination [35], and the Dementia Rating Scale [36]. On the MMSE, the healthy elderly group received a mean score of 28.8 (S.D. = 2.8), or 1.4 standard deviations above normative data [37], and a DRS mean score of 140.3 (S.D. = 2.2), or 0.8 standard deviations above the norm [36]. All MMSE and DRS scores were converted into T scores (M = 50, SD = 10). On both measures, all participants in the healthy elderly group had T scores above the mean of 50, which indicates that all participants were above normative data (see Table 1).

#### Apparatus

**Figure 1:**
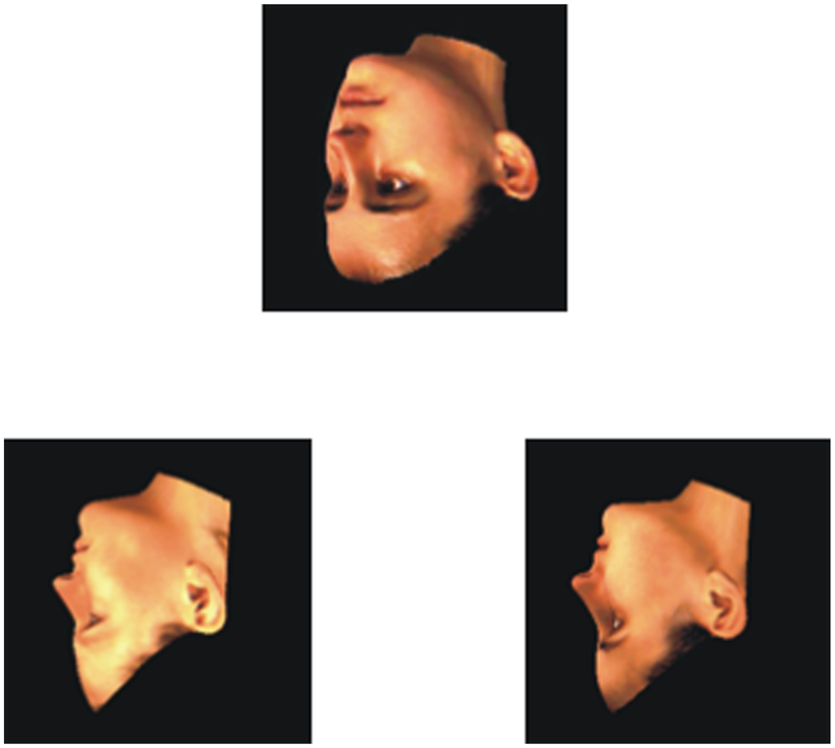
Sample of stimuli used (from Max-Planck Face Database) inverted and rotated in depth around the vertical axis.

The experiment was conducted on a Macintosh PowerBook G4 computer with subjects making their responses on a USB keypad. Stimuli were presented on a 15.4-inch color monitor, positioned approximately 50 cm from the participant, using PsyScope experimental software version 1.2.1 [38]. The stimuli consisted of colour pictures of male and female faces obtained from the Max-Planck Face Database, which contains three–dimensional (3D) models of real faces. Three presentation angles around a vertical axis were selected for the experiments: frontal (F), right three-quarter (T), and right profile (P) (see Figure 1) [8]. The face database was provided by the Max-Planck Institute for Biological Cybernetics in Tuebingen, German. The faces were collected as 3D models and colour maps using a Cyberware™ 3D laser-scanner. Hair was trimmed from the images, leaving the face area alone [39]. Each face was positioned on a black square background (7.5 cm×7.5 cm). A total of 97 faces were used from the database to produce the experimental trials.

Three stimulus faces were presented on each trial. A target face appeared 16.5 cm from the left side of the screen, and 5.5 cm from the top of the screen. Below this face, two choice faces appeared; one was presented on the lower left side of the screen (9.5 cm from left, 16 cm from top), and the other was presented on the lower right side of the screen (22.5 cm from left, 16 cm from top). All three stimuli appeared at once on a gray background. In one set of trials, all stimuli (target and choices) were presented upright; in the other set of trials, all stimuli were inverted. Although the rotation angle of the target face could differ from the rotation angle of the choice faces by 0°, 45°, or 90°, the two choice faces were always rotated to the same presentation angle within a trial (see Figure 1). This resulted in a total of nine possible target×choice face combinations. In three of the combinations (FF, TT, PP) the rotation angle of the target and choice faces did not differ (0°). In four of the combinations the rotation angle between target and choice faces differed by 45° (FT, TF, TP, PT). In two of the combinations, the rotation angle between the target and choice faces differed by 90° (FP, and PF). Participants were presented with 4 blocks (2 blocks of upright, and 2 blocks of inverted trials), consisting of 72 trials each, which were counterbalanced across all subjects.

#### Design and procedure

Each trial began with a fixation cross appearing on the computer screen for 250 ms, followed by the three faces (one on top and two on the bottom). Participants were instructed that their task was to determine which of the two bottom choice faces (left or right) was the same as the top (target) face, regardless of how the pictures were rotated. The stimuli appeared on the screen until a participant made a response or a 10 s time limit had passed, at which point the faces were replaced by a gray screen. After a response had been logged, the experimenter initiated the next trial. The 10 s time limit was chosen because in a pilot study comparing a young and older non-neurological control group, the exposure time for the faces was unlimited and participants in the older group often took an exceedingly long time to respond, without a noticeable benefit in accuracy.

All participants were told to make their responses as quickly, but as accurately as possible. Any responses made after the 10 s time limit, at which point the faces disappeared, were coded as incorrect in order to avoid a reliance on memory. This resulted in 0.53% of the trials for the young participants, and 2.83% of trials for the healthy elderly participants, being coded as incorrect on these grounds.

#### Analysis

Since there were nine possible target×choice face combinations, with three of the combinations (FF, TT, PP) the rotation angle differing by 0°, four of the combinations (FT, TF, TP, PT) differing by 45°, and two of the combinations (FP, PF) differing by 90°, the number of trials was not standard across the different levels of angular disparity. Therefore, the mean percentage of trials that were answered correctly was computed at each level of this variable, for both upright and inverted displays. These mean values were entered into a 2×2×3 [group (older and younger)×planar orientation (upright and inverted)×angular disparity between target and choice face (0°, 45°, 90°)] repeated measures analysis of variance (ANOVA). For all post-hoc comparisons, an alpha level of.05 was adjusted for multiple comparisons using a Bonferroni correction.

### Experiment #2 – The effects of Alzheimer's disease on Face Processing

#### Participants

Nine right-handed participants who were diagnosed with AD by a qualified medical professional (7 males, 2 females, mean age = 85.9, S.D. = 4.9, mean education = 11.7 years, S.D. = 2.7) were recruited from the Alzheimer Society of Manitoba and from personal care homes in Winnipeg, MB. On the MMSE, this AD group received a mean score of 18.3 (S.D. = 4.5), or −4.8 standard deviations below normative data [37], and a mean score of 95.8 (S.D. = 13.2) on the DRS, or −2.4 standard deviations below the norm [36] (see Table 2 and Table S1).

**Table 2:** Information on age, education, MMSE, DRS, and visual acuity scores for the AD group.

Subject	Age	Education (years)	MMSE (raw score)	MMSE (T-score)	DRS (raw score)	DRS (T-score)	Visual acuity	Cataracts
1	78	11	15	−30	86	24	20/30	Yes (both)
2	81	16	22	−5.6	110	28	20/50	Yes (both)
3	80	8	17	7.9	78	24	20/30	No
4	87	12	23	35	93	24	20/30	Yes (both)
5	91	10	18	10	90	24	20/50	Yes (both)
6	90	16	22	11.5	106	28	20/25	No
7	90	10	23	35	111	30.5	20/50	Yes (both)
8	88	10	10	−30	80	24	20/70	Yes (both)
9	88	12	15	−5	108	28	20/25	No

Because our AD group was significantly older than the full sample of older individuals who participated in Experiment 1, their performance was compared to the subset of healthy controls who were age 78 or over (the age of our youngest AD participant). This was deemed necessary, as within the full sample of elderly participants there was a significant negative correlation between age and overall accuracy [*r* = −0.63, *p*<0.05]. This subgroup, hereafter referred to as the “oldest-old” comparison group, consisted of 1 male and 5 females, with a mean age of 82.3 years (SD = 3.9), and a mean of 10.5 years of education (SD = 1.4). An independent samples t-test revealed that the oldest-old control group and the AD group were matched on age [*t*_(13)_ = −1.49, *p*>0.05], and education [*t*_(13)_ = −0.68, *p*>0.05].

#### Design and procedure

The face-matching task of Experiment 2 generally replicated the design and procedure of Experiment 1. The exception was that all but two of the AD participants (Cases 3 and 4) indicated their responses by pointing to the choice face that they felt matched the target face on the screen, rather than entering their responses on the computer keyboard directly. This was done due to motor difficulties using the USB keypad. Once a choice was made, the experimenter entered the response. As in Experiment 1, responses made after the 10 s limit were coded as incorrect; 22.19% of AD participants' responses were coded as incorrect by this criterion. Although we coded responses made after 10 seconds as incorrect, analysis revealed that of these “incorrect” responses, 55.43% of those responses would be correct, with an average response time of 13.04 seconds. From the responses given after the 10 s time limit – 36% of that data required a 45° rotation, 45% required a 90° rotation, while only 19% required no transformation in depth. Since the experimenter responded for most of the AD patients, we felt an analysis of reaction times would not be meaningful. However, reaction times are presented in Table S2 for those interested.

#### Analysis

Most AD participants were only able to comfortably complete two blocks of trials (one upright and one inverted). This meant that, often, the oldest-old participants had completed twice as many trials as the AD participants. To compensate for this, in the analyses described below the scores obtained by the AD group were compared to the scores obtained by the oldest-old participants' performance on the first two blocks of trials only (*n* = 144 trials, in total).

## Results

### Experiment #1 – The effects of aging on face processing

#### Effects of inversion on face recognition

**Figure 2:**
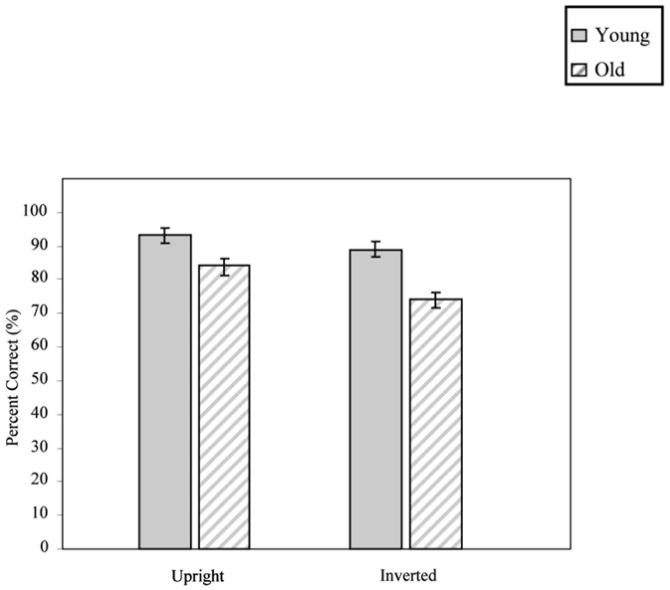
Accuracy under both upright and inverted conditions for both the younger and older viewers (errors bars: SEM's).

The older group was less accurate than the younger group, regardless of planar orientation [*F*_(1,25)_ = 13.70, *p*<0.001]. Despite this, both groups exhibited an effect of stimulus inversion, producing more errors when faces were inverted [*F*_(1,25)_ = 36.15, *p*<0.001]. This effect, however, was more pronounced in the older participants [planar orientation×group, *F*_(1,25)_ = 5.91, *p*<0.05] (see Figure 2).

#### Effects of Increasing Angular Disparity between Target and Choice Faces

As the angular disparity between the target and choice face increased, participants both showed a systematic decrease in accuracy [*F*_(2,50)_ = 47.61, *p*<0.001]. The most accurate responses occurred when no transformation in depth was required (0°), more errors were produced during a 45° rotation, and the most errors were generated when a 90° rotation was required. No interaction was present between rotation angle and group [*F*_(2,50)_ = 0.14, *p*>0.05].

#### Is there a “better” view?

**Figure 3:**
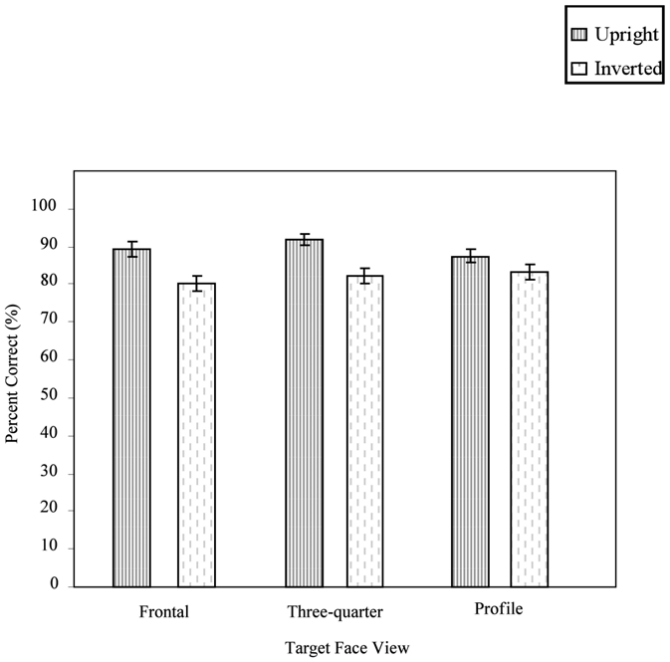
The effects of target face views for both upright and inverted orientation conditions in frontal, three-quarter, and profile views, on older and young adults' ability to match faces (error bars: SEM's).

The mean percent correct for each combination of target and choice faces, in each orientation, were submitted to a 2×2×3×3 [group×planar orientation×target face (frontal, three-quarter, profile)×choice face (frontal, three-quarter, profile)] repeated measures ANOVA. A significant interaction between the target face and choice faces was observed [*F*_(4,100)_ = 31.80, *p*<0.001], and follow-up tests confirmed that the most accurate responses occurred when all faces were presented in the three-quarter view. A significant interaction between planar orientation and target face was also found [*F*_(2,50)_ = 5.00, *p*<0.01]. Follow-up tests on this interaction showed that, while inverting the faces did not impair accuracy for profile views, accuracy was significantly impaired for frontal and three-quarter views (see Figure 3). There were no significant main effects or interactions involving Group.

### Experiment #2 – The effects of Alzheimer's disease on Face Processing

#### Face matching task

**Figure 4:**
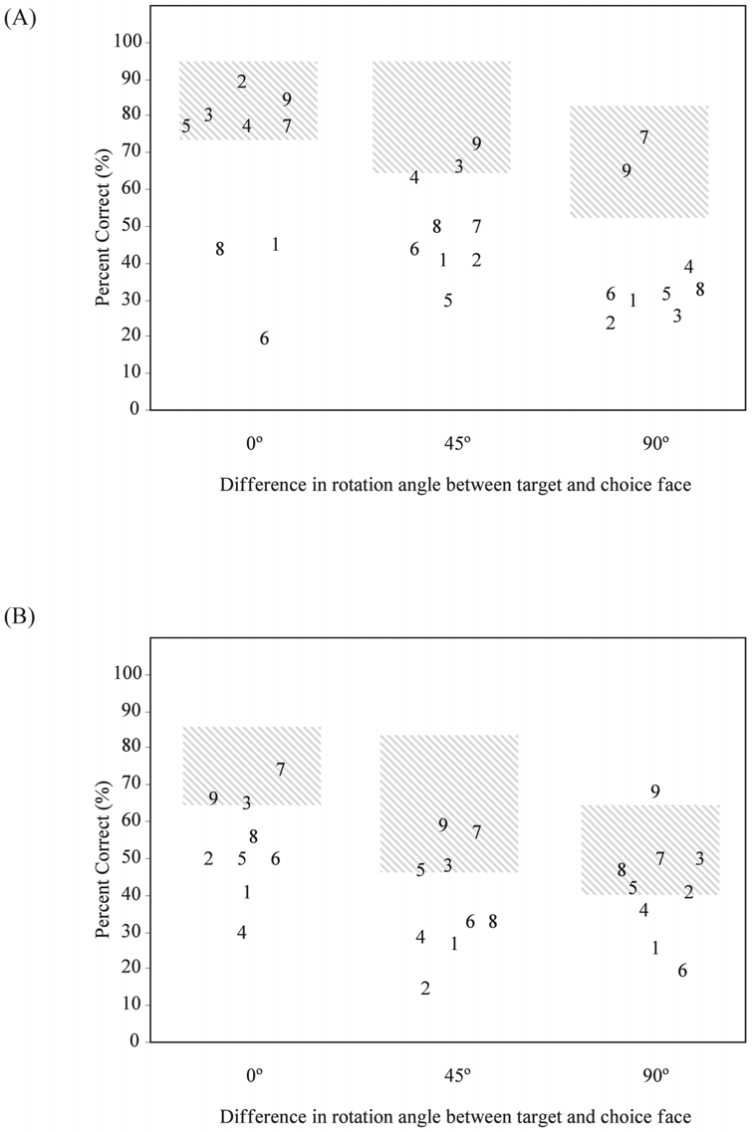
Comparing the impact of increasing the angular disparity between target and choice face angle (0°, 45°, and 90°) for each AD participant (number) in both (a) upright and (b) inverted orientation conditions. Hatched regions represent 95% CIs for the oldest-old control participants (n = 6).

When faces were upright and required no transformation in depth (0°), the majority of AD participants performed within the 95% CI of the oldest-old controls (see Figure 4A, 4B represents inverted faces). However, three participants (1, 6, and 8) were below chance levels in this simple matching condition, which suggests that either they did not understand the task, or that they have a basic perceptual problem that affects their ability to discriminate between faces. For these reasons, they were excluded from subsequent analyses. Group comparisons confirmed that the six remaining AD participants continued to be matched in age and education with the six oldest-old controls.

#### Effects of Inversion and Rotation Angle

**Figure 5:**
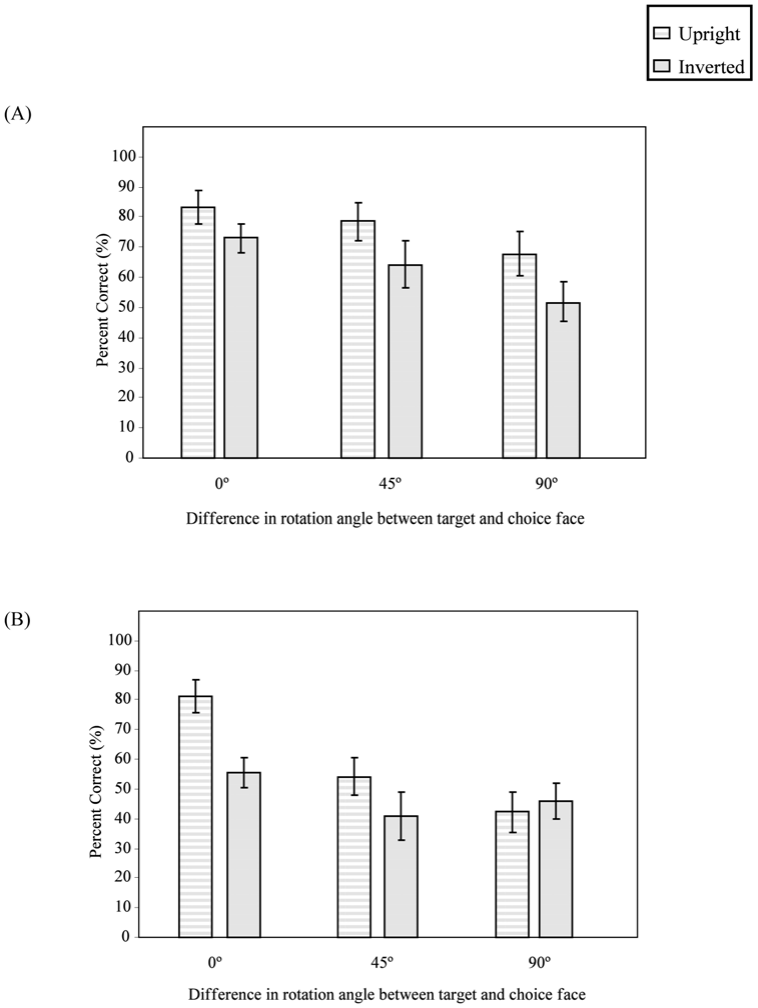
The effects of planar orientation and angular disparity between target and choice face in accuracy for the (A) oldest-old control participants, and (B) for the six AD participants (error bars: SEM's).

The AD participants were significantly less accurate than the oldest-old control group [*F*_(1,10)_ = 5.95, *p*<0.05]. Both groups exhibited the inversion effect, producing more errors when faces were inverted compared to when upright [*F*_(1,10)_ = 30.51, *p*<0.001]. However, a significant three-way interaction was observed between planar orientation, rotation angle, and group [*F*_(2,20)_ = 3.92, *p*<0.05]. In the oldest-old control group, while inverting a face did not impair accuracy for a 0° or a 45° difference, accuracy was significantly impaired when faces were presented at a 90° difference (see Figure 5A). In contrast, for the six AD participants, inverting a face significantly impaired accuracy even in the 0° condition (see Figure 5B). Performance was equally poor with upright and inverted faces when the target and choice faces differed by either 45° or 90°.

#### What “view” works best for AD patients?

**Figure 6:**
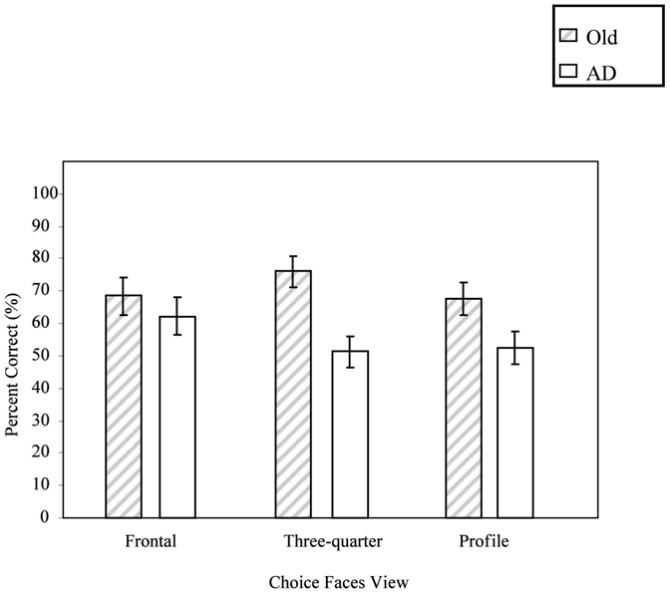
The impact of choice face orientation on matching accuracy for both the oldest-old control participants, and the six AD participants (error bars: SEM's).

The mean percent correct was computed for each combination of target and choice faces, in each orientation. For both groups, a significant interaction between target and choice face was found [*F*_(4,40)_ = 10.40, *p*<0.001], with the most accurate responses occurring when all faces were presented in frontal views. A significant interaction between choice face angle and group was found [*F*_(2,20)_ = 5.92, *p*<0.01]. While for the oldest-old controls, accuracy was not affected by the orientation of the choice faces, the AD group performed significantly better when choice faces were presented in the frontal view (see Figure 6).

#### Correlations

For all nine participants in the AD group, Pearson's bivariate correlations were computed between overall accuracy on the face mental rotation task, the visual acuity scores, and T scores for both the MMSE and the DRS. No significant correlations were found between overall accuracy and visual acuity [*r* = −0.25, *p* = 0.52], between T scores on the DRS and overall accuracy [*r* = 0.04, *p* = 0.93], or between T scores on the MMSE and overall accuracy [*r* = 0.43, *p* = 0.25].

Since the AD participants did poorly with any mental rotation, or inversion, and therefore this could be a reason why no correlations were observed, the same correlations as above were computed with the exception of correlating performance on the upright 0° condition with all specified scales. Once again, no significant correlations were found between accuracy and visual acuity [*r* = −0.19, *p* = 0.62], and between T scores on the DRS and accuracy [*r* = 0.01, *p* = 0.80], between T scores on the MMSE and accuracy [*r* = 0.27, *p* = 0.48].

For the six AD participants that completed the face-matching task, no significant correlations were found between accuracy and visual acuity [*r* = 0.21, *p* = 0.69], between T scores on the DRS and accuracy [*r* = 0.26, *p* = 0.63], or between T scores on the MMSE and accuracy [*r* = −0.35, *p* = 0.55].

## Discussion

The experiments presented here examined the ability of young adults, older non-neurological controls and patients with Alzheimer's disease to complete a time-limited face-matching task in which stimuli could be rotated in depth and/or inverted. We wanted to determine if aging on its own hampers the ability of individuals to mentally rotate faces; if AD patients are disproportionately impaired; if orientation affects young and old participants in the same way and if there was a “preferred” view of the face in which performance was better for each of the groups.

### 

#### Normal Aging

Even though healthy older controls were less accurate on the face-matching task than young adults, both groups exhibited a systematic effect of rotating a face in depth – as the angular disparity between the target and choice faces increased, accuracy decreased. This result has extended the recent finding that healthy older individuals are impaired at making matches with synthetic faces across a 20° rotation from full-face [17]. Here we show that this impairment is present even with real 3D images of faces.

When faces were inverted, the healthy elderly viewers were significantly more impaired than younger controls. Inverting a face disrupts the ability to process it holistically [11], [12], and triggers the adoption of a parts-based analysis. It is possible that the older group had more difficulty switching to a parts-based strategy. This finding coincides with those of Dror et al. [16], who showed that when older adults rotated simple and complex images, a holistic approach was used for both tasks to reduce cognitive load – a pattern not seen with younger viewers. Application of a holistic/configural processing approach may not always serve older viewers well. In fact, recent research has shown that aging results in an impairment in the ability to encode configural information during facial expression recognition [40], [41]. Therefore, when examining age-related changes in holistic processing, it may be very important to consider the particular task demands.

#### Alzheimer's disease

When faces were upright and no mental rotation was involved, the majority of AD participants performed within the 95% CI of the oldest-old control participants. However, participants 1, 6, and 8 performed below chance levels on this simple matching task. It may be that these individuals did not fully understand the task, or they have a basic perceptual problem that affects their ability to discriminate between faces.

For the rest of the AD patients, the fact that they performed comparably with the oldest-old group when no mental rotation was required does support previous findings [28], [29] and suggests that their basic face perception abilities were intact. However, when the target and choice faces differed in orientation or when the stimuli were inverted, the AD group was severely impaired. This lack of a systematic effect of orientation suggests that the underlying difficulties impairing their ability to mentally rotate objects carries over even to “special” stimuli like faces.

Of course, it is also possible that the damage that occurs in Alzheimer's disease to areas like the ventral temporal cortex, which is important in face processing [42], [43], may cause these individuals to rely on processing approaches that do not work well with changes in viewpoint, or face inversion. In short, a form of viewpoint-dependency, where any rotation in depth results in a reduction in accuracy, could be contributing to the AD group's difficulty with matching faces presented at different angles. These results are intriguingly similar to those seen when prosopagnosic patients are run on the same task [44].

Interestingly, in contrast to the young adults and the younger-elderly participants, who both showed a three-quarter view preference, both the oldest-old subgroup and the AD group performed most accurately with frontal views of the target face. It may be that as we age, an increased reliance on holistic processing [16], makes the frontal view more useful for face processing – a valuable piece of information for anyone with an AD patient in their care.

#### Limitations

Even though it is difficult to make strong conclusions based on the small number of AD patients presented in the current investigation, the findings do suggest that it is not memory alone that contributes to AD patients' difficulty with face recognition. Instead, an underlying problem with mental rotation may affect patients ability to process or match any object or face.

Although there was a 10 second time limit for viewing these faces (which was followed by a blank screen), it may be the case that with more time, the AD group could have done better at this task. However, it is also possible that the AD participants equated the blank screen that appeared after 10 seconds as a reminder to respond to the task. Future studies are required to view if an increase in time for viewing the faces would benefit the AD participants.

#### Conclusions

Although face recognition deficits in people with AD have traditionally been associated with memory impairments, our results suggest that an underlying problem with mental rotation may compound these recognition difficulties. This difficulty forming representations that are robust to a variety of spatial transformations appears to occur early in the disease process, so it seems surprising that the literature does not typically report problems with face perception/recognition until later stages of AD [45], [46]. Of course, these reports are often from caregivers who are not conducting the kind of detailed face perception/mental rotation experiments carried out in the current study. It may be that the AD patients' ability to use contextual cues hides this deficit until later stages of AD when the damage has spread to more frontal regions of the brain and they are no longer able to piece together the contextual cues.

## Supporting Information

Table S1 Raw (non-standardized) sub-scores from the DRS for the AD group.
(0.04 MB DOC)

Table S2 Reaction Times
(0.09 MB DOC)
