# Supplementary material for: Mental Rotation of Faces in Healthy Aging and Alzheimer's Disease
Source: PLoS One. 2009 Jul 2;4(7):e6120. doi: 10.1371/journal.pone.0006120 (PMC2700266; doi:10.1371/journal.pone.0006120)
Supplement: Table S1 — Raw (non-standardized) sub-scores from the DRS for the AD group. (0.04 MB DOC) [file pone.0006120.s001.doc]

**Table S1:** Raw (non-standardized) sub-scores from the DRS for the AD group.

| Subject | Attention | Initiation/Perseveration | Construction | Conceptualization | Memory |
| --- | --- | --- | --- | --- | --- |
| 1 | 35 | 19 | 5 | 16 | 11 |
| 2 | 37 | 22 | 6 | 36 | 9 |
| 3 | 24 | 12 | 6 | 22 | 14 |
| 4 | 36 | 17 | 6 | 24 | 10 |
| 5 | 34 | 14 | 6 | 31 | 5 |
| 6 | 31 | 16 | 6 | 34 | 19 |
| 7 | 36 | 19 | 6 | 36 | 14 |
| 8 | 31 | 20 | 6 | 20 | 3 |
| 9 | 33 | 21 | 6 | 36 | 12 |
