# Supplementary material for: Mental Rotation of Faces in Healthy Aging and Alzheimer's Disease
Source: PLoS One. 2009 Jul 2;4(7):e6120. doi: 10.1371/journal.pone.0006120 (PMC2700266; doi:10.1371/journal.pone.0006120)
Supplement: Table S2 — Reaction Times (0.09 MB DOC) [file pone.0006120.s002.doc]

**Table S2: Reaction times**

*Young Participants Reaction Time (RT)*

| **Subject** | **Upright 0º difference between Target and Choice Face RT (ms)** | **Upright 45º difference between Target and Choice Face RT (ms)** | **Upright 90º difference between Target and Choice Face RT (ms)** |
| --- | --- | --- | --- |
| 1 | 1355.24 | 2123.88 | 2966.35 |
| 2 | 1809.08 | 2702.71 | 3800.57 |
| 3 | 1430.44 | 2602.22 | 3138.59 |
| 4 | 1295.75 | 1652.68 | 1875.87 |
| 5 | 1633.40 | 2659.86 | 4308.99 |
| 6 | 1184.71 | 1304.59 | 1395.48 |
| 7 | 1323.96 | 2504.88 | 2662.51 |
| 8 | 1333.29 | 2206.66 | 2621.68 |
| 9 | 1324.02 | 1615.50 | 1997.71 |
| 10 | 1473.19 | 2045.90 | 2979.26 |
| 11 | 1821.34 | 2230.09 | 2397.47 |
| 12 | 963.98 | 1289.40 | 1594.32 |
| 13 | 2025.42 | 2180.59 | 3069.68 |
| 14 | 1084.52 | 1673.79 | 1828.38 |
| 15 | 1809.77 | 2891.69 | 3808.33 |

| **Subject** | **Inverted 0º difference between Target and Choice Face RT (ms)** | **Inverted 45º difference between Target and Choice Face RT (ms)** | **Inverted 90º difference between Target and Choice Face RT (ms)** |
| --- | --- | --- | --- |
| 1 | 2673.05 | 3421.02 | 3922.17 |
| 2 | 2765.35 | 4486.36 | 5424.87 |
| 3 | 1985.13 | 2421.76 | 3188.50 |
| 4 | 1523.23 | 1807.33 | 2219.23 |
| 5 | 1717.19 | 2418.29 | 3145.44 |
| 6 | 1523.84 | 1465.00 | 1676.27 |
| 7 | 1746.14 | 2610.51 | 2409.21 |
| 8 | 2132.01 | 2150.01 | 2630.77 |
| 9 | 1947.03 | 2548.86 | 3002.81 |
| 10 | 2451.07 | 3496.85 | 3488.49 |
| 11 | 2971.16 | 3413.29 | 3121.17 |
| 12 | 1305.57 | 1385.78 | 1987.19 |
| 13 | 1819.52 | 2162.86 | 2191.41 |
| 14 | 1819.58 | 2586.67 | 2625.81 |
| 15 | 2504.58 | 3521.27 | 4415.15 |

# Healthy Elderly Adults RT

| Subject | **Upright 0º difference between Target and Choice Face RT (ms)** | **Upright 45º difference between Target and Choice Face RT (ms)** | **Upright 90º difference between Target and Choice Face RT (ms)** |
| --- | --- | --- | --- |
| 1 | 3618.67 | 5395.69 | 5638.05 |
| 2 | 2219.02 | 2996.49 | 3672.89 |
| 3 | 1955.98 | 2422.38 | 2834.57 |
| 4 | 3336.61 | 4178.18 | 4495.98 |
| 5 | 3568.86 | 4341.27 | 5231.47 |
| 6 | 2490.09 | 3294.63 | 3720.08 |
| 7 | 1913.27 | 2860.17 | 3425.91 |
| 8 | 4709.56 | 5210.21 | 6563.83 |
| 9 | 2648.58 | 3442.46 | 4496.92 |
| 10 | 2150.29 | 2430.35 | 3013.07 |
| 11 | 2263.64 | 2968.24 | 3739.58 |
| 12 | 2798.57 | 4564.90 | 6197.31 |

| Subject | **Inverted 0º difference between Target and Choice Face RT (ms)** | **Inverted 45º difference between Target and Choice Face RT (ms)** | **Inverted 90º difference between Target and Choice Face RT (ms)** |
| --- | --- | --- | --- |
| 1 | 4936.30 | 6540.59 | 7638.88 |
| 2 | 3426.05 | 4272.81 | 5154.69 |
| 3 | 2092.48 | 2878.57 | 3378.03 |
| 4 | 3968.77 | 5348.33 | 5531.62 |
| 5 | 3379.97 | 3995.87 | 4606.26 |
| 6 | 4108.25 | 4018.55 | 5147.75 |
| 7 | 3064.31 | 4209.11 | 5103.70 |
| 8 | 5285.29 | 5715.24 | 5650.02 |
| 9 | 4513.69 | 5007.40 | 6951.03 |
| 10 | 2983.42 | 2891.07 | 3459.28 |
| 11 | 2739.54 | 3199.27 | 4464.29 |
| 12 | 3131.62 | 4133.82 | 5051.54 |

# Alzheimer’s Disease Participants RT

| Subject | **Upright 0º difference between Target and Choice Face RT (ms)** | **Upright 45º difference between Target and Choice Face RT (ms)** | **Upright 90º difference between Target and Choice Face RT (ms)** |
| --- | --- | --- | --- |
| 1 | 4220.29 | 4303.88 | 5343.53 |
| 2 | 4297.50 | 5132.05 | 5663.81 |
| 3 | 5215.56 | 5194.06 | 6310.13 |
| 4 | 5386.67 | 5978.67 | 6692.94 |
| 5 | 6017.09 | 6139.92 | 6786.88 |
| 6 | 6272.30 | 6243.50 | 6981.67 |
| 7 | 6544.86 | 6925.25 | 7247.20 |
| 8 | 6713.09 | 7350.10 | 7743.75 |
| 9 | 7563.06 | 8192.38 | 7782.71 |

| Subject | **Inverted 0º difference between Target and Choice Face RT (ms)** | **Inverted 45º difference between Target and Choice Face RT (ms)** | **Inverted 90º difference between Target and Choice Face RT (ms)** |
| --- | --- | --- | --- |
| 1 | 4256.25 | 3231.00 | 4096.38 |
| 2 | 4508.73 | 4241.14 | 5189.33 |
| 3 | 5337.63 | 4912.57 | 5297.13 |
| 4 | 5498.12 | 4983.75 | 5325.60 |
| 5 | 5530.57 | 5569.23 | 5532.33 |
| 6 | 5996.10 | 6058.20 | 6261.00 |
| 7 | 6124.14 | 6387.13 | 6290.33 |
| 8 | 6471.42 | 8188.36 | 7824.82 |
| 9 | 8093.75 | 8425.40 | 9034.89 |
